# Maternal Separation Induces Sex-Specific Differences in Sensitivity to Traumatic Stress

**DOI:** 10.3389/fnbeh.2021.766505

**Published:** 2021-12-10

**Authors:** Dayan Knox, Stephanie A. Stout-Oswald, Melissa Tan, Sophie A. George, Israel Liberzon

**Affiliations:** ^1^Department of Psychological and Brain Sciences, University of Delaware, Newark, DE, United States; ^2^Department of Psychiatry, University of Michigan, Ann Arbor, MI, United States; ^3^Veterans Affairs Hospital, Ann Arbor, MI, United States; ^4^Department of Psychiatry and Behavioral Neurosciences, Wayne State University, Detroit, MI, United States; ^5^Department of Psychiatry and Behavioral Sciences, Texas A&M University, Bryant, TX, United States

**Keywords:** anxiety, sex differences, PTSD - posttraumatic stress disorder, maternal care, startle

## Abstract

Post-traumatic stress disorder (PTSD) is a debilitating psychiatric disorder with a high economic burden. Two risk factors for increasing the chances of developing PTSD are sex (being female) and early life stress. These risk factors suggest that early life stress-induced changes and sex differences in emotional circuits and neuroendocrinological systems lead to susceptibility to traumatic stress. Exploring mechanisms *via* which stress leads to specific effects can be accomplished in animal models, but reliable animal models that allow for an examination of how early life stress interacts with sex to increase susceptibility to traumatic stress is lacking. To address this, we examined the effects of early life stress [using the maternal separation (MS) model] and late adolescence/early adult traumatic stress [using the single prolonged stress (SPS) model] on startle reactivity, anxiety-like behavior in the open field (OF), and basal corticosterone levels in male and female rats. Female rats exposed to MS and SPS (MS/SPS) showed enhanced startle reactivity relative to MS/control female rats. Enhanced startle reactivity was not observed in MS/SPS male rats. Instead, non-maternally separated male rats that were exposed to SPS showed enhanced startle reactivity relative to controls. Female rats had enhanced locomotor activity in the OF and higher basal corticosterone levels in comparison to males, but measures in the OF and basal corticosterone were not affected by MS or SPS. Overall the results suggest that the combined MS and SPS models can be used to explore how changes in maternal care during infancy lead to sex differences in sensitivity to the effects of traumatic stress as adolescents and adults.

## Introduction

Post-traumatic stress disorder (PTSD) is a debilitating disorder that has a prevalence rate of 7.8% of the US population (Kessler et al., 1995), and the socioeconomic cost associated with PTSD is extensive (Kessler, 2000; Dams et al., 2020). Identifying neurobiological mechanisms *via* which traumatic stress leads to PTSD symptoms is important. To this end, animal models can be useful. One such model is single prolonged stress (SPS), which refers to serial exposure to restraint, forced swim, and ether (Yamamoto et al., 2009; Bowers and Ressler, 2015; Deslauriers et al., 2018). Key SPS effects that model PTSD symptoms include enhanced fast negative feedback of the HPA axis, arousal, and re-experiencing symptoms (Liberzon et al., 1997, 1999; Khan and Liberzon, 2004; Kohda et al., 2007; George et al., 2012, 2015; Knox et al., 2012). SPS also is relatively easy to implement and can be adapted in a range of research settings (Ferland-Beckham et al., 2021). However–like many animal models of PTSD—SPS was developed in male animals and the consistent effects observed in these animals are not often observed in female animals (Bowers and Ressler, 2015; Keller et al., 2015; Deslauriers et al., 2018; Pooley et al., 2018a, b). Because women are more likely to develop PTSD after trauma exposure than men (Li and Graham, 2017; Hodes and Epperson, 2019; Christiansen and Berke, 2020), establishing animal models that recapitulate this sex difference is important.

Early life stress refers to acute, chronic, and/or traumatic stressful experiences that occur *in utero* through adolescence (Horn et al., 2016; Turecki and Meaney, 2016; Jiang et al., 2019) and may increase the chances of developing PTSD in men and women (Horn et al., 2016; Turecki and Meaney, 2016; Jiang et al., 2019). Maternal separation (MS) is a validated model of early life stress that induces effects on emotional circuits and neuroendocrinological systems that have been well characterized in rats and also observed in humans. These include blunted hypothalamic-pituitary-adrenal (HPA) axis reactivity, reduction in glucocorticoid receptor (GR) expression in key emotional substrates, and increased anxiety (Liu et al., 1997; Caldji et al., 1998, 2000; Francis and Meaney, 1999; Francis et al., 1999; Weaver et al., 2004; Turecki and Meaney, 2016). MS effects are varied but consistently reported, in female model systems (for examples see de Jongh et al., 2005; Wei et al., 2018; Cui et al., 2020; Farinetti et al., 2020). In most studies utilizing animal models of PTSD, early life stress is rarely considered as a factor but could be critical in determining how traumatic stress effects manifest in adolescent and adult animals. Previous studies have combined MS and SPS to examine how MS in neonates modulates SPS effects in adults. In previous reports, MS enhanced the effects of SPS on anxiety-like behavior in male and female rats (Imanaka et al., 2006; Sun et al., 2021) and contextual conditioned freezing and analgesia in male rats (Imanaka et al., 2006). Another study observed that early life stress could build resilience to anxiogenic effects and working memory deficits induced by SPS (Yang et al., 2019). However, no study to date has examined how MS and SPS affect core PTSD symptoms (e.g., enhanced arousal) in male and female rats.

To address this, we examined the effects of MS and SPS on the baseline and light-enhanced startle (measures of arousal), anxiety-like behavior in the open field (OF), and basal corticosterone levels (a measure of HPA axis activity) in male and female rats.

## Materials and Methods

### Animals

Ninety-six Sprague-Dawley rats across 12 litters were included in this study. All rats were maintained on a 12 h:12 h light-dark cycle and housed in a temperature-controlled (21 ± 1°C) and humidity-controlled (55 ± 5%) environment with *ad libitum* access to standard rat chow and water. Timed-pregnant dams were obtained from Charles River Inc. (Portage, MI) and arrived at the Ann Arbor Veterans Affairs Veterinary Medical Center at approximately gestation day 16. Dams were housed singly in clean cages with nesting material and sawdust for the duration of the pregnancy and post-natal weaning period. Day of birth was marked postnatal day (PND) 0. All animal procedures were approved by the institutional review board in the Ann Arbor Veterans Affairs Medical Center and in accordance with National Institute of Health Guide for the Care and Use of Laboratory Animals (NIH Publications No. 80 23, revised 1978).

### Neonatal and Late Adolescent/Early Adult Stress

On PND 2, litters were culled to 4–12 pups to obtain even numbers of males and females in each litter, though most litters ranged from 8 to 12 pups with equal (or near equal) numbers of males and females. Each litter was then randomly assigned to one of two early stress conditions: MS or animal facility rearing (AFR). A variation of previously published MS protocols (Ladd et al., 2004; Aisa et al., 2008) was carried out daily from PND 2–23 between 12:00 and 15:00 during the light cycle. For MS, each litter was removed from the home cage for 180 min daily over a 3-week period. Litters were placed in a nest of sawdust and nesting material in a clean cage that was maintained at approximately 32°C by an electric heating pad beneath the cage. Dams were left undisturbed in the home cage. AFR control litters were left undisturbed except for bi-weekly cage changes. After completion of MS at PND 23, all pups were weaned and caged in same sex littermate pairs.

On PND 37 male and female rats were subjected to SPS, separately, as previously described (Liberzon et al., 1997; Knox et al., 2010). Within all litters, an equivalent number of male and female rats were randomly assigned to the SPS or control groups. Animals were restrained for 120 min, then in groups of six-eight animals were subjected to forced swim for 20 min and ether until general anesthesia was induced. At the same time, another group of rats was taken to a novel room and left in this room for the duration of SPS. These animals served as the control (i.e., no SPS) group. After SPS, 7 days were allowed to elapse before commencing behavioral tests. This post-stress incubation period is necessary in order to observe SPS effects (Liberzon et al., 1997, 1999; Knox et al., 2012).

### Behavioral Tests and Corticosterone Assays

At PND 45 animals were subjected to the OF test followed the next day by the light-enhanced startle test. The dimensions of the OF arena were 91.5 cm width × 91.5 cm length × 61 cm height. The walls of the OF arena were opaque and the floor of the OF arena was transparent with a grid drawn on the floor dividing it into 25 segments (approximately 19 cm width × 19 cm length). For behavioral scoring, the arena was divided into an outer region (15.3 cm from the walls) and inner region (61 cm × 61 cm). After a 10-min acclimation period to the room housing the OF arena, each rat was placed in the center of the OF and allowed to explore it for 5 min. The OF was cleaned with 70% ethanol between each individual test. OF behavior was recorded using a camera and scored at a later date. Behavioral measures in the OF test were scored by individuals blind to the group assignment of rats. Open space avoidance was measured by calculating the time spent in, and entries made into, the inner region of the field, while segment crossings were used as a measure of locomotor activity. A segment crossing was defined when more than three-quarters of a rat’s body entered into a neighboring segment. Time spent and entries made in the outer region of the OF were also recorded.

The light-enhanced acoustic startle procedure was conducted on the second testing day as previously described (Walker and Davis, 1997; Khan and Liberzon, 2004). Following a 5-min acclimation period, the procedure was carried out in a single session in a dimly illuminated room using the SR-LAB system (San Diego Instruments, San Diego, CA). Rats were placed in a Plexiglas cylinder atop a piezoelectric accelerometer, which transduced movement into voltage deflections. Plexiglas cylinders were contained within a ventilated sound-attenuated chamber. Throughout the session, background white noise at 55 dB was interrupted by 30 startle tones (50 ms duration, 100 dB intensity, near-instantaneous rise time) presented at varying intervals of 25–35 s. After each startle tone presentation, the activity of the accelerometer was recorded for up to 20 ms. Presentation of startle tones and recording of voltage deflections were controlled automatically using SR-LAB (San Diego Instruments). Startle tones and background noise were calibrated using an audiometer (RadioShack). All animals received three sessions of startle tone presentation at baseline, and in light and dark environments. The first set of startle tones was always conducted in the dark and was used to establish baseline levels of startle reactivity. Startle tone presentation was then repeated in the presence of a bright light (>400 Lux) or in the dark again. After this second set of startle tones, there was a third presentation of tones that was either presented in the light or the dark. Thus, every animal had its baseline startle response established, and then light and dark startle responses were measured in the second and third sets of startle tone presentations. In total, all animals received three sessions of startle tone presentations (e.g., baseline, light, dark). The light and dark startle sessions were counterbalanced across all rats.

Four days after the light-enhanced startle session rats were euthanized and trunk blood collected in EDTA coated tubes, then centrifuged at 1,000× *g* for 20 min. Plasma was collected and stored at −80°C until the time of assay. Corticosterone was assayed using a corticosterone kit (tkrc1) in accordance with the manufacturer’s instructions (Siemens, Los Angeles CA).

### Data and Statistical Analysis

Behavioral measures in the OF were subjected to a sex (male vs. female) × neonatal stress (MS vs. AFR) × late adolescent/early adult (LA/EA) stress (SPS vs. control) factor design. A startle response was defined as the maximum voltage deflection that occurred 10 ms after the onset of a startle tone. Startle responses were subjected to a sex × neonatal stress × LA/EA stress × trial (baseline, light, dark) factor design. Baseline corticosterone levels were subjected to a sex × neonatal stress × LA/EA stress factor design. Main and simple effects were analyzed using analysis of variance while main and simple comparisons were analyzed using least square differences (LSD) tests. The criterion for significance was set a *p* < 0.05. All statistical tests were conducted using IBM SPSS statistics version 28.

## Results

**Figure 1:**
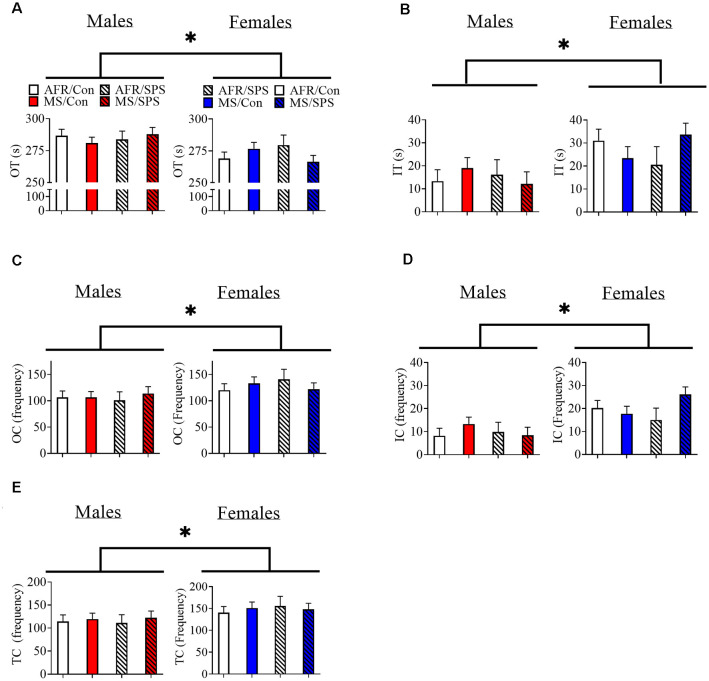
The effects of MS and SPS on OF behavior. **(A,B)** Female rats (AFR/control = 10, AFR/SPS = 4, MS/control = 10, MS/SPS = 10) spent less time in the outer region **(A)** and more time in the inner region **(B)** of the OF arena than male rats (AFR/control = 10, AFR/SPS = 12, MS/control = 6, MS/SPS = 9). However, **(C–E)** female rats made more entries into the inner and outer regions of the OF arena. Neither MS nor SPS had any statistically significant effect on OF behavior. *Statistical comparison that has a *p* < 0.05. OT, outer time; IT, inner time; OC, outer crossing; IC, inner crossing; TC, total crossing; MS, maternal separation; SPS, single prolonged stress; AFR, animal facility rearing; OF, open field.

Female rats (*n* = 34) spent less time in the outer region (and more time in the inner region) of the OF in comparison to male rats (*n* = 37). Statistics for these variables are identical, because they are the inverse of each other (animals are either in the inner or outer regions) [*F*_(1,63)_ = 9.024, *p* = 0.004; Figures 1A,B]. There was a sex × neonatal stress × LA/EA stress interaction that approached significance [*F*_(1,63)_ = 3.644, *p* = 0.061]. This trend effect may have reflected the finding that male rats exposed to MS and SPS (*n* = 10) spent more time in the outer region of the OF, while female rats exposed to MS and SPS (*n* = 10) spent less time in the outer region of the OF (Figure 1A). Complimentary findings were observed for time spent in the inner region of the OF, where female rats spent more time in the inner region of the OF [*F*_(1,63)_ = 9.024, *p* = 0.004; Figure 1B], but this effect may have been strongest in female rats exposed to MS and SPS [*F*_(1,63)_ = 3.644, *p* = 0.061; see Figure 1B]. Female rats (*n* = 34) made more outer [*F*_(1,63)_ = 5.088, *p* = 0.028; see Figure 1C] and inner [*F*_(1,63)_ = 14.122, *p* < 0.001; see Figure 1D] segment crossings than male rats (*n* = 37) and also made more total crossings than male rats [*F*_(1,63)_ = 8.296, *p* = 0.005; see Figure 1E].

**Figure 2:**
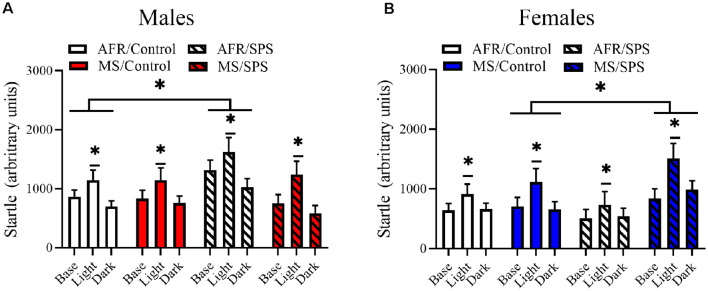
The effects of MS and SPS on startle reactivity. All rats showed light-enhanced startle. **(A)** Male rats (AFR/control = 17, AFR/SPS = 8, MS/control = 8, MS/SPS = 10) from the AFR group showed enhanced startle reactivity after SPS exposure and this effect was absent in male SPS rats that were subjected to MS as neonates. **(B)** Female rats (AFR/control = 18, AFR/SPS = 10, MS/control = 10, MS/SPS = 8) from the AFR treatment did not show enhanced startle reactivity to SPS, but females rats from the MS group showed enhanced startle reactivity after exposure to SPS. *Significant statistical test at *p* < 0.05.

**Figure 3:**
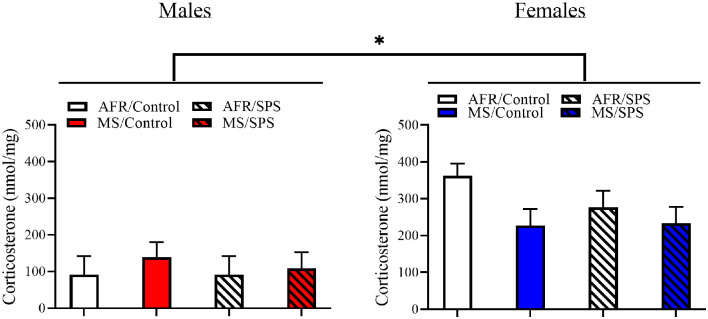
The effects of MS and SPS on basal corticosterone levels. Female rats (AFR/control = 18, AFR/SPS = 10, MS/control = 10, MS/SPS = 10) had higher levels of corticosterone than males (AFR/control = 18, AFR/SPS = 8, MS/control = 12, MS/SPS = 10), but basal corticosterone levels were not affected by MS or SPS. *Significant statistical test at *p* < 0.05.

All rats demonstrated light-enhanced startle [main effect of trial: *F*_(2,172)_ = 44.282, *p* < 0.001]. There was a sex × neonatal stress × LA/EA stress interaction [*F*_(1,86)_ = 4.769, *p* = 0.032]. This effect was driven by the finding that female rats exposed to MS and SPS (*n* = 8) had enhanced startle relative to female rats exposed to MS, but not SPS (*n* = 10), but male rats that had no early life stress (i.e., AFR) and SPS (*n* = 8) had enhanced startle relative to male controls (i.e., no MS or SPS, *n* = 17). This interpretation was supported by simple comparisons for SPS vs. control for MS/female rats [Difference—516.51 ± 226.904; *F*_(1,86)_ = 5.182, *p* = 0.025] and simple comparisons for SPS vs. control for AFR/male rats [Difference—418.108 ± 203.262; *F*_(1,86)_ = 4.231, *p* = 0.043]. These results are illustrated in Figure 2. There were no significant stress effects on basal corticosterone levels (*p*s > 0.05) in male or female rats, but basal corticosterone levels were enhanced in female rats (*n* = 48) relative to male rats (*n* = 48) [*F*_(1,88)_ = 26.801, *p* < 0.001]. These results are illustrated in Figure 3.

## Discussion

In a replication of previous findings, we observed that SPS enhanced startle reactivity/arousal in male rats that were subject to AFR (Khan and Liberzon, 2004; Kohda et al., 2007; George et al., 2012). This effect was not observed in female rats that were subject to AFR. MS altered the impact of SPS on startle reactivity in a sex-specific manner. SPS enhanced startle reactivity in female rats exposed to MS but had no effect on male rats exposed to MS as neonates. These results suggest that MS, at least with regards to startle reactivity and arousal, produces a sex-specific shift in sensitivity to traumatic stress in LA/EAs where female rats exposed to MS become sensitive to traumatic stress in LA/EA, but male rats exposed to MS become resilient.

Studies have demonstrated that early life stress renders male and female humans more likely to develop PTSD as adults (Bremner et al., 1993; Breslau et al., 1999; Chapman et al., 2004; Anda et al., 2006; Cougle et al., 2010; Dunn et al., 2014). However, early life stress is typically defined across a broad developmental period (infancy to adolescence) and the selective effect of impoverished mother-infant interactions on future susceptibility to traumatic stress as adults has not been examined. Because the MS paradigm models impoverished maternal care (Caldji et al., 1998, 2000; Francis and Meaney, 1999; Francis et al., 1999; Weaver et al., 2004), the results of this study raise the possibility that MS-induced changes in mother-female pup interactions may lead to sensitivity to traumatic stress as animals mature, but MS-induced changes in mother-male pup interactions may lead to resilience to traumatic stress as animal mature. Alternatively, MS-induced changes in mother-pup interactions may have differential effects on stress resilience in male and female animals. Female rats mature at a faster rate than male rats and the time at which SPS was applied may have corresponded to a different phase of adolescence in males vs. females. This raises the additional possibility that maternal care during the neonatal phase of development produced sex-dependent differences in sensitivity to traumatic stress by changing the emergence of adolescent-related neuroendocrinological phenomena. PTSD is more prevalent in women than men (Li and Graham, 2017; Hodes and Epperson, 2019; Christiansen and Berke, 2020). Because the results of this study raise the possibility that disrupted maternal care in infancy could lead to sex differences in sensitivity to traumatic stress in adulthood, more research examining the possible role of infant maternal care in sex differences in PTSD is needed.

How might MS render female rats sensitive to the negative effects of SPS, but not have the same effect in males? MS can alter the properties of genes that control the emotional circuits in the brain *via* behavioral and epigenetic mechanisms. MS decreases maternal licking, grooming, and arched back nursing of pups and is associated with enhanced DNA methylation of exon 1_7_ of the Nr3c1 gene (Francis et al., 1999; Weaver et al., 2004; Turecki and Meaney, 2016) and subsequent decreases in GR expression in the HPA axis, hippocampus, and medial prefrontal cortex (Liu et al., 1997; Francis and Meaney, 1999; Francis et al., 1999; Weaver et al., 2004; van der Doelen et al., 2014; Turecki and Meaney, 2016). Similar effects have been observed in humans subjected to childhood abuse (Turecki and Meaney, 2016; Jiang et al., 2019). Together these findings raise the possibility that MS-induced changes in GR function in substrates that regulate startle and arousal (e.g., locus coeruleus, bed nucleus of the stria terminalis; Pardon et al., 2002) could underlie sex differences in susceptibility to traumatic stress in adults.

In this study, MS (without subsequent stressors) had no effect on startle reactivity or anxiety behavior in the OF in male and female rats. These results are consistent with previous reports that suggest MS has no effect on baseline startle reactivity (Lehmann et al., 2000; de Jongh et al., 2005; Kao et al., 2012; Llido et al., 2013), though other reports have observed enhancements in startle reactivity with MS (Kalinichev et al., 2002; Groenink et al., 2011). There are consistent reports of MS enhancing anxiety-like behavior generated by open space in rodents (Caldji et al., 2000; Huot et al., 2001, 2002; Kalinichev et al., 2002; Llido et al., 2013; Cui et al., 2020). Changes in specific maternal behavior (e.g., changes in licking behavior vs. arched back nursing) induced by the separation protocol used in this study as well as the strain of rat used (Long-Evans vs. Sprague Dawley) could account for the differences in anxiety-like behavior observed in this study and other reports. Another possibility is the timing at which MS was performed in dams. In this study, MS was performed between 12 and 3 pm, which was delayed in time from the light cycle for these rats. Other studies perform MS close to the start of the light cycle (for example see de Jongh et al., 2005). This relatively late start for MS could have driven maternal behaviors that had no impact on anxiety-like behavior.

Both MS and SPS are associated with changes in HPA axis function. MS results in higher corticotrophin-releasing hormone levels in the HPA axis and lower GR levels in key regions of the HPA axis a substrate that regulates HPA axis activity (Liu et al., 1997; Caldji et al., 1998, 2000; Francis and Meaney, 1999; Francis et al., 1999; Weaver et al., 2004; Turecki and Meaney, 2016). SPS enhances fast-negative feedback of the HPA axis (Liberzon et al., 1997, 1999), dorsal hippocampus and mPFC GR expression (Eagle et al., 2013; George et al., 2015), and stress-induced internalization of amygdala GRs (Moulton et al., 2018). We observed the well-established finding that females have higher levels of basal corticosterone than males (Kitay, 1961; Bangasser and Wicks, 2017), but neither MS nor SPS were associated with changes in baseline corticosterone levels. This finding suggests that characterizing the effects of MS and SPS on HPA axis function requires methods that can measure dynamic changes in HPA axis activity (e.g., fast-negative feedback, glucocorticoid receptor internalization) rather than basal hormonal measures.

Avoidance of open space is often used to model anxiety in rodents. We observed that female rats spent more time in the inner region of the OF, which could be interpreted as an anxiolytic effect. However, female rats had increased locomotion in the OF in comparison to male rats (see “Results” section). Differences in locomotor activity between males and females in novel open spaces may represent a confound and have to be carefully addressed when using the OF (or any behavioral test that uses open space) to measure sex differences in anxiety reactivity in rats.

## Conclusion

The results of the study suggest that changes in early life maternal care can generate sex differences in susceptibility to traumatic stress in adolescence and adulthood. Specifically, we observed that MS rendered female rats sensitive to traumatic stress, but rendered male rats resilient to the same type of traumatic stress. Changes in methylation of Nr3C1 have been consistently implicated in early life stress effects, but other genes that regulate norepinephrine, serotonin, BDNF, and regulators of GR activity (e.g., CRH, FKBP5) could also play a role in the sex-specific effect MS has on sensitivity to adult traumatic stress (Wang et al., 2018; Jiang et al., 2019; Sun et al., 2021). Other salient variables, such as potential shifts in the estrous cycle induced by MS, need to be considered as well. Further research is needed to elucidate mechanisms by which variations in neonatal care (including manipulations that enhance maternal care such as early handling) result in sex differences in sensitivity to the effects of traumatic stress on startle reactivity and arousal.

## Data Availability Statement

The raw data supporting the conclusions of this article will be made available by the authors, without undue reservation.

## Ethics Statement

The animal study was reviewed and approved by IUCAC committee of the University of Delaware.

## Author Contributions

DK: helped edit and write the manuscript, conduct experiments, and analyze data. SS-O: helped write the manuscript and conduct experiments. MT and SG: helped edit the manuscript and conduct experiments. IL: designed experiments and edited manuscript. All authors contributed to the article and approved the submitted version.

## Conflict of Interest

The authors declare that the research was conducted in the absence of any commercial or financial relationships that could be construed as a potential conflict of interest.

## Publisher’s Note

All claims expressed in this article are solely those of the authors and do not necessarily represent those of their affiliated organizations, or those of the publisher, the editors and the reviewers. Any product that may be evaluated in this article, or claim that may be made by its manufacturer, is not guaranteed or endorsed by the publisher.

## References

[B1] Aisa B. Tordera R. Lasheras B. Del Rio J. Ramirez M. J. (2008). Effects of maternal separation on hypothalamic-pituitary-adrenal responses, cognition and vulnerability to stress in adult female rats. Neuroscience 154, 1218–1226. 10.1016/j.neuroscience.2008.05.011 18554808

[B2] Anda R. F. Felitti V. J. Bremner J. D. Walker J. D. Whitfield C. Perry B. D.. (2006). The enduring effects of abuse and related adverse experiences in childhood. A convergence of evidence from neurobiology and epidemiology. Eur. Arch. Psychiatry Clin. Neurosci. 256, 174–186. 10.1007/s00406-005-0624-4 16311898 PMC3232061

[B3] Bangasser D. A. Wicks B. (2017). Sex-specific mechanisms for responding to stress. J. Neurosci. Res. 95, 75–82. 10.1002/jnr.23812 27870416 PMC5120612

[B4] Bowers M. E. Ressler K. J. (2015). An overview of translationally informed treatments for posttraumatic stress disorder: animal models of pavlovian fear conditioning to human clinical trials. Biol. Psychiatry 78, E15–27. 10.1016/j.biopsych.2015.06.008 26238379 PMC4527085

[B5] Bremner J. D. Southwick S. M. Johnson D. R. Yehuda R. Charney D. S. (1993). Childhood physical abuse and combat-related posttraumatic stress disorder in vietnam veterans. Am. J. Psychiatry 150, 235–239. 10.1176/ajp.150.2.235 8422073

[B6] Breslau N. Chilcoat H. D. Kessler R. C. Davis G. C. (1999). Previous exposure to trauma and PTSD effects of subsequent trauma: results from the detroit area survey of trauma. Am. J. Psychiatry 156, 902–907. 10.1176/ajp.156.6.902 10360130

[B7] Caldji C. Francis D. Sharma S. Plotsky P. M. Meaney M. J. (2000). The effects of early rearing environment on the development of GABAA and central benzodiazepine receptor levels and novelty-induced fearfulness in the rat. Neuropsychopharmacology 22, 219–229. 10.1016/S0893-133X(99)00110-4 10693149

[B8] Caldji C. Tannenbaum B. Sharma S. Francis D. Plotsky P. M. Meaney M. J. (1998). Maternal care during infancy regulates the development of neural systems mediating the expression of fearfulness in the rat. Proc. Natl. Acad. Sci. U S A 95, 5335–5340. 10.1073/pnas.95.9.5335 9560276 PMC20261

[B9] Chapman D. P. Whitfield C. L. Felitti V. J. Dube S. R. Edwards V. J. Anda R. F. (2004). Adverse childhood experiences and the risk of depressive disorders in adulthood. J. Affect Disord. 82, 217–225. 10.1016/j.jad.2003.12.013 15488250

[B10] Christiansen D. M. Berke E. T. (2020). Gender- and sex-based contributors to sex differences in PTSD. Curr. Psychiatry Rep. 22:19. 10.1007/s11920-020-1140-y 32125541

[B11] Cougle J. R. Timpano K. R. Sachs-Ericsson N. Keough M. E. Riccardi C. J. (2010). Examining the unique relationships between anxiety disorders and childhood physical and sexual abuse in the national comorbidity survey-replication. Psychiatry Res. 177, 150–155. 10.1016/j.psychres.2009.03.008 20381878

[B12] Cui Y. Cao K. Lin H. Cui S. Shen C. Wen W.. (2020). Early-life stress induces depression-like behavior and synaptic-plasticity changes in a maternal separation rat model: gender difference and metabolomics study. Front. Pharmacol. 11:102. 10.3389/fphar.2020.00102 32174832 PMC7055479

[B13] Dams J. Rimane E. Steil R. Renneberg B. Rosner R. Konig H. H. (2020). Health-related quality of life and costs of posttraumatic stress disorder in adolescents and young adults in germany. Front. Psychiatry 11:697. 10.3389/fpsyt.2020.00697 32760304 PMC7373788

[B14] de Jongh R. Geyer M. A. Olivier B. Groenink L. (2005). The effects of sex and neonatal maternal separation on fear-potentiated and light-enhanced startle. Behav. Brain Res. 161, 190–196. 10.1016/j.bbr.2005.02.004 15878207

[B15] Deslauriers J. Toth M. Der-Avakian A. Risbrough V. B. (2018). Current status of animal models of posttraumatic stress disorder: behavioral and biological phenotypes and future challenges in improving translation. Biol. Psychiatry 83, 895–907. 10.1016/j.biopsych.2017.11.019 29338843 PMC6085893

[B16] Dunn E. C. Solovieff N. Lowe S. R. Gallagher P. J. Chaponis J. Rosand J.. (2014). Interaction between genetic variants and exposure to hurricane katrina on post-traumatic stress and post-traumatic growth: a prospective analysis of low income adults. J. Affect Disord. 152–154, 243–249. 10.1016/j.jad.2013.09.018 24161451 PMC3873605

[B17] Eagle A. L. Knox D. Roberts M. M. Mulo K. Liberzon I. Galloway M. P.. (2013). Single prolonged stress enhances hippocampal glucocorticoid receptor and phosphorylated protein kinase B levels. Neurosci. Res. 75, 130–137. 10.1016/j.neures.2012.11.001 23201176 PMC3604057

[B18] Farinetti A. Aspesi D. Marraudino M. Marzola E. Amianto F. Abbate-Daga G.. (2020). Sexually dimorphic behavioral effects of maternal separation in anorexic rats. Dev. Psychobiol. 62, 297–309. 10.1002/dev.21909 31502241

[B19] Ferland-Beckham C. Chaby L. E. Daskalakis N. P. Knox D. Liberzon I. Lim M. M.. (2021). Systematic review and methodological considerations for the use of single prolonged stress and fear extinction retention in rodents. Front. Behav. Neurosci. 15:652636. 10.3389/fnbeh.2021.652636 34054443 PMC8162789

[B20] Francis D. Diorio J. Liu D. Meaney M. J. (1999). Nongenomic transmission across generations of maternal behavior and stress responses in the rat. Science 286, 1155–1158. 10.1126/science.286.5442.1155 10550053

[B21] Francis D. D. Meaney M. J. (1999). Maternal care and the development of stress responses. Curr. Opin. Neurobiol. 9, 128–134. 10.1016/s0959-4388(99)80016-6 10072372

[B22] George S. A. Knox D. Curtis A. Valentino R. Liberzon I. (2012). Altered locus coeruleus-norepinephrine function following single prolonged stress. Eur. J. Neurosci. 37, 901–909. 10.1111/ejn.12095 23279008

[B23] George S. A. Rodriguez-Santiago M. Riley J. Rodriguez E. Liberzon I. (2015). The effect of chronic phenytoin administration on single prolonged stress induced extinction retention deficits and glucocorticoid upregulation in the rat medial prefrontal cortex. Psychopharmacology (Berl) 232, 47–56. 10.1007/s00213-014-3635-x 24879497

[B24] Groenink L. Bijlsma E. Y. van Bogaert M. J. Oosting R. S. Olivier B. (2011). Serotonin1A receptor deletion does not interact with maternal separation-induced increases in startle reactivity and prepulse inhibition deficits. Psychopharmacology (Berl) 214, 353–365. 10.1007/s00213-010-1998-1 20811879 PMC3045511

[B25] Hodes G. E. Epperson C. N. (2019). Sex differences in vulnerability and resilience to stress across the life span. Biol. Psychiatry 86, 421–432. 10.1016/j.biopsych.2019.04.028 31221426 PMC8630768

[B26] Horn S. R. Charney D. S. Feder A. (2016). Understanding resilience: new approaches for preventing and treating PTSD. Exp. Neurol. 284, 119–132. 10.1016/j.expneurol.2016.07.002 27417856

[B27] Huot R. L. Plotsky P. M. Lenox R. H. McNamara R. K. (2002). Neonatal maternal separation reduces hippocampal mossy fiber density in adult Long Evans rats. Brain Res. 950, 52–63. 10.1016/s0006-8993(02)02985-2 12231228

[B28] Huot R. L. Thrivikraman K. V. Meaney M. J. Plotsky P. M. (2001). Development of adult ethanol preference and anxiety as a consequence of neonatal maternal separation in long evans rats and reversal with antidepressant treatment. Psychopharmacology (Berl) 158, 366–373. 10.1007/s002130100701 11797057

[B29] Imanaka A. Morinobu S. Toki S. Yamawaki S. (2006). Importance of early environment in the development of post-traumatic stress disorder-like behaviors. Behav. Brain Res. 173, 129–137. 10.1016/j.bbr.2006.06.012 16860405

[B30] Jiang S. Postovit L. Cattaneo A. Binder E. B. Aitchison K. J. (2019). Epigenetic modifications in stress response genes associated with childhood trauma. Front. Psychiatry 10:808. 10.3389/fpsyt.2019.00808 31780969 PMC6857662

[B31] Kalinichev M. Easterling K. W. Plotsky P. M. Holtzman S. G. (2002). Long-lasting changes in stress-induced corticosterone response and anxiety-like behaviors as a consequence of neonatal maternal separation in long-evans rats. Pharmacol. Biochem. Behav. 73, 131–140. 10.1016/s0091-3057(02)00781-5 12076732

[B32] Kao G. S. Cheng L. Y. Chen L. H. Tzeng W. Y. Cherng C. G. Su C. C.. (2012). Neonatal isolation decreases cued fear conditioning and frontal cortical histone 3 lysine 9 methylation in adult female rats. Eur. J. Pharmacol. 697, 65–72. 10.1016/j.ejphar.2012.09.040 23051673

[B33] Keller S. M. Schreiber W. B. Staib J. M. Knox D. (2015). Sex differences in the single prolonged stress model. Behav. Brain Res. 286, 29–32. 10.1016/j.bbr.2015.02.034 25721741 PMC5745062

[B34] Kessler R. C. (2000). Posttraumatic stress disorder: the burden to the individual and to society. J. Clin. Psychiatry 5, 4–12. 10761674

[B35] Kessler R. C. Sonnega A. Bromet E. Hughes M. Nelson C. B. (1995). Posttraumatic stress disorder in the national comorbidity survey. Arch. Gen. Psychiatry 52, 1048–1060. 10.1001/archpsyc.1995.03950240066012 7492257

[B36] Khan S. Liberzon I. (2004). Topiramate attenuates exaggerated acoustic startle in an animal model of PTSD. Psychopharmacology (Berl) 172, 225–229. 10.1007/s00213-003-1634-4 14586539

[B37] Kitay J. I. (1961). Sex differences in adrenal cortical secretion in the rat. Endocrinology 68, 818–824. 10.1210/endo-68-5-818 13756461

[B38] Knox D. George S. A. Fitzpatrick C. J. Rabinak C. A. Maren S. Liberzon I. (2012). Single prolonged stress disrupts retention of extinguished fear in rats. Learn Mem. 19, 43–49. 10.1101/lm.024356.111 22240323 PMC3262971

[B40] Knox D. Perrine S. A. George S. A. Galloway M. P. Liberzon I. (2010). Single prolonged stress decreases glutamate, glutamine and creatine concentrations in the rat medial prefrontal cortex. Neurosci. Lett. 480, 16–20. 10.1016/j.neulet.2010.05.052 20546834 PMC2902659

[B41] Kohda K. Harada K. Kato K. Hoshino A. Motohashi J. Yamaji T.. (2007). Glucocorticoid receptor activation is involved in producing abnormal phenotypes of single-prolonged stress rats: a putative post-traumatic stress disorder model. Neuroscience 148, 22–33. 10.1016/j.neuroscience.2007.05.041 17644267

[B42] Ladd C. O. Huot R. L. Thrivikraman K. V. Nemeroff C. B. Plotsky P. M. (2004). Long-term adaptations in glucocorticoid receptor and mineralocorticoid receptor mRNA and negative feedback on the hypothalamo-pituitary-adrenal axis following neonatal maternal separation. Biol. Psychiatry 55, 367–375. 10.1016/j.biopsych.2003.10.007 14960289

[B43] Lehmann J. Stohr T. Feldon J. (2000). Long-term effects of prenatal stress experiences and postnatal maternal separation on emotionality and attentional processes. Behav. Brain Res. 107, 133–144. 10.1016/s0166-4328(99)00122-9 10628737

[B44] Li S. H. Graham B. M. (2017). Why are women so vulnerable to anxiety, trauma-related and stress-related disorders? The potential role of sex hormones. Lancet Psychiatry 4, 73–82. 10.1016/S2215-0366(16)30358-3 27856395

[B45] Liberzon I. Abelson J. L. Flagel S. B. Raz J. Young E. A. (1999). Neuroendocrine and psychophysiologic responses in PTSD: a symptom provocation study. Neuropsychopharmacology 21, 40–50. 10.1016/S0893-133X(98)00128-6 10379518

[B46] Liberzon I. Krstov M. Young E. A. (1997). Stress-restress: effects on ACTH and fast feedback. Psychoneuroendocrinology 22, 443–453. 10.1016/s0306-4530(97)00044-9 9364622

[B47] Liu D. Diorio J. Tannenbaum B. Caldji C. Francis D. Freedman A.. (1997). Maternal care, hippocampal glucocorticoid receptors and hypothalamic-pituitary-adrenal responses to stress. Science 277, 1659–1662. 10.1126/science.277.5332.1659 9287218

[B48] Llido A. Modol L. Darbra S. Pallares M. (2013). Interaction between neonatal allopregnanolone administration and early maternal separation: effects on adolescent and adult behaviors in male rat. Horm. Behav. 63, 577–585. 10.1016/j.yhbeh.2013.02.002 23410958

[B49] Moulton E. Chamness M. Knox D. (2018). Characterizing changes in glucocorticoid receptor internalization in the fear circuit in an animal model of post traumatic stress disorder. PLoS One 13:e0205144. 10.1371/journal.pone.0205144 30532228 PMC6286002

[B50] Pardon M. C. Gould G. G. Garcia A. Phillips L. Cook M. C. Miller S. A.. (2002). Stress reactivity of the brain noradrenergic system in three rat strains differing in their neuroendocrine and behavioral responses to stress: implications for susceptibility to stress-related neuropsychiatric disorders. Neuroscience 115, 229–242. 10.1016/s0306-4522(02)00364-0 12401336

[B51] Pooley A. E. Benjamin R. C. Sreedhar S. Eagle A. L. Robison A. J. Mazei-Robison M. S.. (2018a). Sex differences in the traumatic stress response: PTSD symptoms in women recapitulated in female rats. Biol. Sex Differ. 9:31. 10.1186/s13293-018-0191-9 29976248 PMC6034295

[B52] Pooley A. E. Benjamin R. C. Sreedhar S. Eagle A. L. Robison A. J. Mazei-Robison M. S.. (2018b). Sex differences in the traumatic stress response: the role of adult gonadal hormones. Biol. Sex Differ. 9:32. 10.1186/s13293-018-0192-8 30001741 PMC6043950

[B53] Sun H. Zhang X. Kong Y. Gou L. Lian B. Wang Y.. (2021). Maternal separation-induced histone acetylation correlates with BDNF-programmed synaptic changes in an animal model of PTSD with sex differences. Mol. Neurobiol. 58, 1738–1754. 10.1007/s12035-020-02224-6 33245480

[B54] Turecki G. Meaney M. J. (2016). Effects of the social environment and stress on glucocorticoid receptor gene methylation: a systematic review. Biol. Psychiatry 79, 87–96. 10.1016/j.biopsych.2014.11.022 25687413 PMC4466091

[B55] van der Doelen R. H. Calabrese F. Guidotti G. Geenen B. Riva M. A. Kozicz T.. (2014). Early life stress and serotonin transporter gene variation interact to affect the transcription of the glucocorticoid and mineralocorticoid receptors and the co-chaperone FKBP5, in the adult rat brain. Front. Behav. Neurosci. 8:355. 10.3389/fnbeh.2014.00355 25352794 PMC4195371

[B56] Walker D. L. Davis M. (1997). Double dissociation between the involvement of the bed nucleus of the stria terminalis and the central nucleus of the amygdala in startle increases produced by conditioned versus unconditioned fear. J. Neurosci. 17, 9375–9383. 10.1523/JNEUROSCI.17-23-09375.1997 9364083 PMC6573581

[B57] Wang Q. Shelton R. C. Dwivedi Y. (2018). Interaction between early-life stress and FKBP5 gene variants in major depressive disorder and post-traumatic stress disorder: a systematic review and meta-analysis. J. Affect Disord. 225, 422–428. 10.1016/j.jad.2017.08.066 28850857 PMC5626653

[B58] Weaver I. C. Cervoni N. Champagne F. A. D’Alessio A. C. Sharma S. Seckl J. R.. (2004). Epigenetic programming by maternal behavior. Nat. Neurosci. 7, 847–854. 10.1038/nn1276 15220929

[B59] Wei Y. Wang G. Wang H. He J. Zhang N. Wu Z.. (2018). Sex-dependent impact of different degrees of maternal separation experience on OFT behavioral performances after adult chronic unpredictable mild stress exposure in rats. Physiol. Behav. 194, 153–161. 10.1016/j.physbeh.2018.04.034 29723593

[B60] Yamamoto S. Morinobu S. Takei S. Fuchikami M. Matsuki A. Yamawaki S.. (2009). Single prolonged stress: toward an animal model of posttraumatic stress disorder. Depress. Anxiety 26, 1110–1117. 10.1002/da.20629 19918929

[B61] Yang R. Sun H. Wu Y. Lu G. Wang Y. Li Q.. (2019). Long-lasting sex-specific effects based on emotion- and cognition-related behavioral assessment of adult rats after post-traumatic stress disorder from different lengths of maternal separation. Front. Psychiatry 10:289. 10.3389/fpsyt.2019.00289 31231246 PMC6558979

